# Comprehensive mutation detection of *BRCA1/2* genes reveals large genomic rearrangements contribute to hereditary breast and ovarian cancer in Chinese women

**DOI:** 10.1186/s12885-019-5765-3

**Published:** 2019-06-07

**Authors:** Wen-Ming Cao, Ya-Bing Zheng, Yun Gao, Xiao-Wen Ding, Yan Sun, Yuan Huang, Cai-Jin Lou, Zhi-Wen Pan, Guang Peng, Xiao-Jia Wang

**Affiliations:** 1 Department of Medical Oncology, Zhejiang Cancer Hospital, 1 Banshan East Road, Hangzhou, 310022 China; 2 Institute of Cancer Research, Zhejiang Cancer Hospital, Hangzhou, 310022 China; 3 Department of Breast Cancer Surgery, Zhejiang Cancer Hospital, Hangzhou, 310022 China; 4 Department of Clinical Laboratory, Zhejiang Cancer Hospital, Hangzhou, 310022 China; 5 Department of Clinical Cancer Prevention, the University of Texas, MD Anderson Cancer Center, Houston, TX 77030 USA

**Keywords:** Chinese, Familial breast cancer, Familial ovarian cancer, *BRCA1*, *BRCA2*, Rearrangement

## Abstract

**Background:**

Mutated *BRCA1/2* genes are associated with hereditary breast and ovarian cancer (HBOC). So far most of the identified *BRCA1/2* pathogenic variants are single nucleotide variants (SNVs) or insertions/deletions (Indels). However, large genomic rearrangements (LGRs) such as copy number variants (CNVs) are also playing an important role in HBOC predisposition. Their frequency and spectrum have been well studied in western populations but remain largely unknown for Chinese population.

**Methods:**

Peripheral blood samples were collected from 218 unrelated familial breast and/or ovarian cancer (FBOC) patients living in Eastern China. PCR-based Sanger sequencing and panel-based next-generation sequencing (NGS) were performed to detect pathogenic SNVs and Indels in BRCA1/2 genes. For the patients lacking small pathogenic variants, multiplex ligation dependent probe amplification (MLPA) assay was conducted to screen for LGRs.

**Results:**

In total, we identified 44 samples (20.1%) carrying small pathogenic variants (26 in *BRCA1* and 18 in *BRCA2,* respectivel*y*). Among the rest of 174 samples, five were found carrying novel deleterious LGRs in *BRCA1* which are exon5-7dup (1 patient), exon13-14dup (2 patients), and exon1-22del (2 patients). No LGR was found in *BRCA2*. Overall, LGRs accounted for 16.1% (5/31) of *BRCA1* pathogenic variants, and were detected in 2.3% (5/218) of all FBOC patients.,

**Conclusions:**

LGR variants in BRCA1 gene play a significant role in Chinese HBOC patients. MLPA or other similar LGR-detecting methods should be recommended along with nucleotide sequencing as the initial screening approach for Chinese HBOC women.

**Electronic supplementary material:**

The online version of this article (10.1186/s12885-019-5765-3) contains supplementary material, which is available to authorized users.

## Background

According to National Central Cancer Registry of China, breast cancer ranks No.1 in cancer incidence and sixth in cancer-associated death for Chinese women, with over 250,000 newly diagnosed cases and 70,000 breast cancer-associated death in 2015 [1]. The average onset age of breast cancer is 45–55 years old for Chinese women, which is also younger than observed for Caucasian women [2]. While majority of breast cancer cases are sporadic, patients with familial history or other risk factors such as early onset age have been frequently observed in clinic, suggesting an important role of genetic factors in the disease development. Indeed, germline pathogenic variants in the two major breast cancer susceptibility genes *BRCA1/2* have been detected within Chinese patients [3–9].

Studying *BRCA1/2* pathogenic variants requires accurate and comprehensive testing methods. Short-read DNA sequencing methods, including both Sanger and next-generation sequencing (NGS), are only capable of reliably detecting small variants such as single nucleotide variants (SNVs) or insertion/ deletion (Indels), but not suitable for detecting large genomic rearrangements (LGRs), which involve deletions or duplications of multiple exons [i.e. copy number variants (CNVs)]. Therefore, sequencing alone may lead to underestimated frequency of pathogenic variants. Southern blotting could be used to detect LGRs [10], but is labor intensive and generally low-throughput. SNP or CGH arrays can detect copy number variants but their unit cost is high and resolution is usually over hundreds of Kb. Several multiplex PCR-based techniques have been recently developed to achieve higher processing and cost efficiency. For instance, multiplex ligation dependent probe amplification (MLPA) assay and multiplex amplicon quantification (MAQ) have been developed as fast and reproducible methods for CNV detection [11]. At the present, MLPA remains to be the most commonly used method for LGRs, and has detected 82.7 and 53% LGRs in *BRCA1* and *BRCA2*, respectively [12].

As of today *BRCA1/2* LGR studies have been mostly conducted in western countries, showing different prevalence with ethnicity and geography. For example, there was no *BRCA1/2* LGR variants detected in Ashkenazi Jewish familial breast cancer patients [13, 14], but in non-Ashkenazi Jewish, the frequency of LGRs was 6% [14]. Very limited research has been conducted for Chinese, and only 12 *BRCA1/2* LGRs have been so far reported. Those studies were conducted in Hong Kong [15], Singapore [16] and Malaysian [17]. The frequency and spectrum of *BRCA1/2* LGRs in familial breast cancer patients from China mainland remain largely unknown.

## Methods

### Patient subjects

A total of 218 unrelated familial breast cancer patients were enrolled into this study between 2008 and 2017. All patients were diagnosed in Zhejiang Cancer Hospital in Eastern China and had a family history of at least one first- or second-degree relatives affected with breast cancer and/or ovarian cancer, regardless of age. Peripheral blood samples from the patients were collected in EDTA tubes and stored at − 80 °C. SNVs and Indels variants of *BRCA1/2* were firstly determined for all patients using sequencing methods (PCR-based Sanger sequencing and panel-based NGS). The patients with negative sequencing finding were further screened for LGRs by MLPA. The written informed consents were obtained from all participating patients prior to clinical data and peripheral blood collection. This study was approved by the Research and Ethical Committee of Zhejiang Cancer Hospital, China. All experiments were performed in accordance with the approved guidelines.

### DNA extraction

Genomic DNA was extracted from peripheral blood samples using QIAamp DNA Blood Mini kit (Qiagen, Hilden, Germany) by following the manufacture’s manual. DNA purity and concentration were measured by NanoDrop 2000 Spectrophotometer and Qubit 3.0 (Thermo Fisher Scientific, Waltham, USA), and DNA integrity was determined by agrose gel electrophoresis.

### Nucleotide sequencing

The present study used both PCR-based Sanger sequencing and panel-based NGS to interrogate small nucleotide variants including SNVs and Indels. In the first phase of the project, Sanger sequencing was performed on 133 unrelated FBOC cases using a total of 72 pairs of oligos to cover all coding exons and intron-exon boundaries of *BRCA1/2*. The primer oligo sequences were listed in Additional file 1: Table S1 and Additional file 2: Table S2. In the second phase of the project, in order to achieve high-throughput and cost-effective sequencing, we designed a NGS panel by adopting the NEBNext Direct sequencing technology developed by New England Biolabs (Ipswich, MA). The panel contains *BRCA1/2* genes as well as other 96 known cancer risk-associated genes. We performed panel NGS on all of the 133 Sanger cases along with 85 new cases newly collected. Individually prepared libraries were pooled for Hiseq X sequencing (Illumina, CA, USA) to achieve a minimum 500x mean coverage for the included panel genes. Raw FASTQ data run through in house bioinformatic pipeline with variant calling generated for *BRCA1/2* genes. Variant filtering and final interpretation were conducted by following the ACMG Standards and Guideline for the Interpretation of Sequence Variants [18] and based on a set of criteria such as allele frequency as well as information from clinical genome databases including ClinVar (https://www.ncbi.nlm.nih.gov/clinvar/), Online Mendelian Inheritance in Man (OMIM) (http://www.omim.org/) and Human Gene and Mutation Database (HGMD) (http://www.hgmd.cf.ac.uk/ac/index.php).

### LGRs analysis

*BRCA1/2* LGRs was screened by (Multiplex ligation dependent probe assay) MLPA assay using the SALSA P002 kit and P045 kit for *BRCA1* and *BRCA2* genes, respectively (MRC-Holland, Amsterdam, the Netherlands). The MLPA reactions were performed according to the manufacturer’s instruction. Five normal control samples were included as reference within each MLPA run. Fragment analysis of the PCR products were performed on an ABI 3130xl Genetic Analyzer (Applied Biosystems, Foster City, CA). The data was analyzed by using the Coffalyser software v.9 (Applied Biosystems, Foster City, CA). All of the peak heights were normalized, and the ratio value between 0.7–1.3 was considered as normal. A ratio value ≤0.7 or ≥ 1.3 was threshold suggestive of a deletion or duplication, respectively. All patients with a value ≤0.7 or ≥ 1.3 were confirmed by independent experiments.

Two primer oligos were designed to validate *BRCA1* Exon 5–7 duplication. The forward primer sequence was CCGTGCCAAAAGACTTCTACA (Exon 7) and the reverse primer sequence was TTGCTTCCAACCTAGCATCA (Exon 5). Long range PCR amplification was performed with Takara LA Taq DNA polymerase (Takara Bio, USA) by following the manufacturer’s manual. The amplified product was run on 0.8% Agrose gel electrophoresis with EB (i.e. ethidium bromide) and visualized under UV light. The purified amplicons were subjected to Sanger sequencing to confirm amplification fidelity.

## Results

### Small pathogenic variants in *BRCA1/2* genes

Overall, we identified a total of 31 *BRCA1* or *BRCA2* pathogenic SNVs and Indels in 44 unrelated patients by combining Sanger sequencing and the 98-gene panel NGS assay. Table 1 lists all these small variants. In summary, nearly 59% (26 of 44) of patients had *BRCA1* pathogenic variants, and 41% (18 of 44) had *BRCA2* pathogenic variants. Two recurrent pathogenic variants (c.5154G > A and c.5468-1_5474del GCAATTGG) in *BRCA1* were reported as putative founder mutations [19]. In total, frequency of *BRCA1/2* small pathogenic variants was 20.2% (44/218) in the studied cohort.

**Table 1:** Small pathogenic variants of *BRCA1* and *BRCA2* in 218 familial breast and/or ovarian cancer patients

Gene	Mutation	AA change	ClinVar	No. of patient	Tumor type (age Dx)	IHC of BC	History of BC and OC (age Dx)	Other cancers in the family (age Dx)
BRCA1	c.223G > T	p.Glu75Ter	No	1	IDC (R 44, L 54)	ER−/PR−/HER2-	S OC(52)	PA EC, PA LC, MU GC
	c.1209delT	p.Glu404Asnfs	No	1	IDC (47), OC, TC	ER+/PR+/HER2-	M OC(52), MA OC(54)	
	c.1465G > T	p.Glu489Ter	Yes	1	IDC (51)	ER−/PR−/HER2-	S OC(52) and BC(58)	
	c.1945G > T	p.Glu649Ter	Yes	1	IDC (36)	ER−/PR−/HER2-	M BC(58)	
	c.2110_2111delAA	p.Asn704Cysfs	Yes	2	IDC (55)	ER−/PR−/HER2-	M BC(57)	
					IDC (68)	ER−/PR−/HER2-	S OC, D OC(40)	
	c.3266delA	p.Leu1089Cysfs	Yes	1	IDC (62)	ER−/PR−/HER2-	S OC(45)	S LC(65),
	c.3295delC	p.Pro1099Leufs0	No	1	IDC (29)	ER−/PR−/HER2-	S BC(40)	
	c.3780_3781delAG	p.Leu1260Phefs	No	2	OC (57)	ND	M BC(58)	S GbC(70)
					IDC (39), OC (44)	NA	MA BC(42), MA BC(33)	
	c.4063_4066delAATC	p.Asn1355Lysfs	No	1	IDC (40)	ER+/PR+/HER2-	M BC(46)	
	c.4065_4068delTCAA	p.Asn1355Lysfs	Yes	2	IDC (38), OC (45)	ER−/PR−/HER2-	M BC (69)	MA EC
					IDC (50)	ER−/PR−/HER2-	S BC	P GC
	c.5154G > A	p.Trp1718Ter	Yes	2	IDC(35)	ER−/PR−/HER2-	S BC(40)	F BT
					IDC (41)	ER−/PR−/HER2-	S BC(45)	M EC(69), MA RC
	c.5161C > T	p.Gln1721Ter	Yes	1	IDC (32)	ER−/PR−/HER2-	S BC(L 35, R 37), M OC(47)	
	c.5173insA	p.Glu1725Argfs	Yes	1	IDC (42)	ER−/PR−/HER2-	S BC(48), MA BC(48)	M LC(51)
	c.5251C > T	p.Arg1751Ter	Yes	1	IDC (47)	ND	S DCIS(57)	
	c.5467 + 1G > A	–	Yes	1	IDC (31)	ER+/PR+/HER2+	S BC(41), S BC(45), MA BC(51)	
	c.5468-1_5474del GCAATTGG	–	No	2	IDC (41)	ER−/PR−/HER2-	S BC(52), S OC(47)	M EC(77), F LuC(81)
					IDC (36)	ER−/PR−/HER2-	S BC(L 38, R 44), MA BC(48)	MA EC(50)
	c.5470_5477del ATTGGGCA	p.Ile1824Aspfs	Yes	5	IDC (36)	ER−/PR−/HER2-	S BC (L 37, R 39)	
					IDC (40)	ER−/PR−/HER2-	M BC(44)	
					IDC (58)	ER−/PR−/HER2-	M OC(55), MA BC(56)	
					IDC (L 22, R 33), TC (22)	ER−/PR−/HER2-	M BC(47), MGM BC(49), MA BC(33), MA OC(42)	MA RC(47)
					IDC (49)	ER−/PR−/HER2-	S BC(52), PA BC	
BRCA2	c.-39-1_-39delGA	–	Yes	1	IDC (46)	ER+/PR+/HER2+	S BC(48)	
	c.469_473delAAGTC	p.Lys157Serfs	No	1	IDC (46)	ER−/PR+/HER2-	S BC(L 47, R 49)	S CC(50), F EsC(51), B EsC(64)
	c.470_474del AGTCA	p.Lys157Serfs	Yes	1	ILC (31)	ER+/PR+/HER2-	M BC(58)	
	c.755_758delACAG	p.Asp252Valfs	Yes	1	IDC (48)	ER+/PR+/HER2-	M OC(68)	
	c.784delG	p.Ala262Glnfs	No	1	IDC (43)	ER+/PR+/HER2-	S BC(43), PA BC(50)	F EsC(57), FU PC(56)
	c.3109C>T	p.Gln1037Ter	Yes	2	IDC (L 39, R 47)	ER+/PR+/HER2-	M BC (39)	
					IDC (34)	ER+/PR+/HER2-	PA BC(51)	PA GC(70), PU EsC (59)
	c.3189_3192delGTCA	p.Ser1064Leufs	Yes	1	IDC (55)	ER+/PR+/HER2-	S BC(55)	
	c.3596_3599delACTG	p.Asp1199Valfs	Yes	1	IDC (44)	NA	S BC(46), M BC(60)	
	c.4487delC	p.Pro1496Glnfs	No	1	IDC (37)	ER−/PR+/HER2-	S BC(47), MS BC(41)	MA TC(51), MA TC(55), MS TC(36)
	c.5495delC	p.Ser1832Leufs	No	1	IDC (41)	ER−/PR+/HER2-	S BC(43)	
	c.5682C > G	p.Tyr1894Ter	Yes	4	ILC (32)	ER+/PR+/HER2-	PA BC(45), PA BC(R 42, L 46)	
					MBC (42)	NA	PGF BC(66)	
					ILC (68)	ER+/PR+/HER2-	PA BC(60)	
					ILC (61)	ER+/PR+/HER2-	S BC(51)	
	c.6141 T > A	p.Tyr2047Ter	No	1	IDC (35)	NA	S BC(39), MGM BC(61), MS BC(50)	S LuC (52), MU LC(66)
	c.6359C > G	p.Ser2120Ter	Yes	1	ILC (R 36, L 51), EC (55)	ER+/PR+/HER2-	M OC(76), MA BC(70)	
	c.7588C > T	p.Gln2530Ter	No	1	IDC (43)	ER+/PR+/HER2+	M BC(63)	

*AA* amino acid, *Dx* diagnosis, *IHC* immunohistochemistry, *IDC* invasive ductal carcinoma, *ILC* invasive lobular carcinoma, *MBC* medullary breast carcinoma, *L* left, *R* right, *BC* breast cancer, *OC* ovarian cancer, *LC* liver cancer, *EC* endometrial carcinoma, *LuC* lung cancer, *BT* brain tumor, *RC* rectal cancer, *DCIS* ductal carcinoma in situ, *GbC* Gallbladder cancer, *GC* gastric cancer, *TC* thyroid cancer, *CC* Colon cancer, *EsC* Esophagus cancer, *PA* prostate cancer, *M* mother, *S* sister, *MS* maternal sister, *PA* paternal aunt, *MGM* maternal grandmother, *F* father, *MU* maternal uncle, *FU* father uncle, *MA* maternal aunt, *D* daughter, *PGF* paternal grandfather, *B* brother, *PU* paternal uncle, *ND* not done, *NA* not available

### Novel LGRs identified in *BRCA1*

**Fig. 1:**
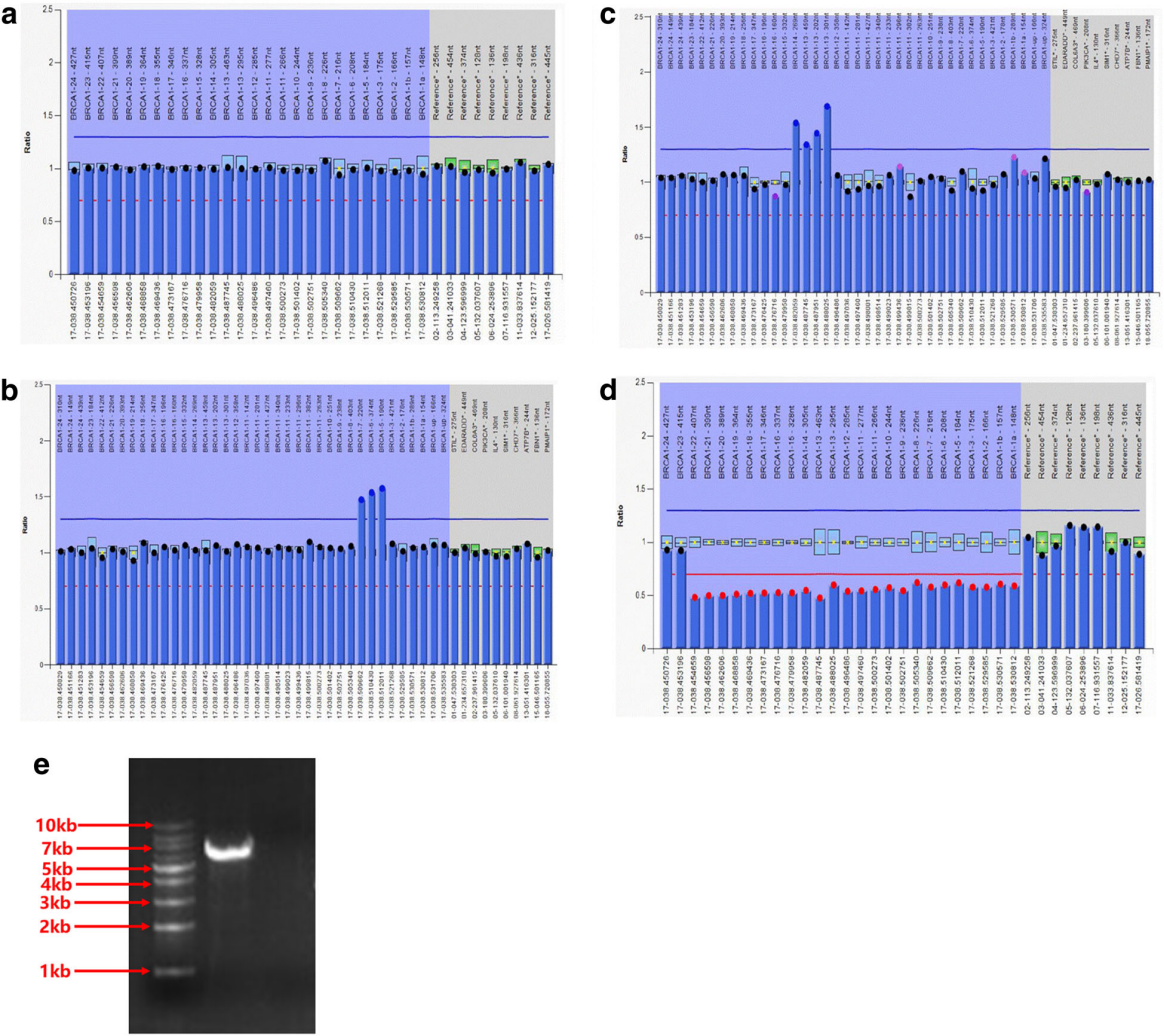
Three *BRCA1* LGRs detected in the HBOC cohort. MLPA result for *BRCA1* LGRs in bar chart format generated by Coffalyser software v.9. *BRCA1* exons and intra normalized ratio are given on the X axis and Y axis, respectively. Exons having reduced or increased peak ratio are denoted by red or blue dot, respectively. **a** MLPA result for *BRCA1* wildtype. **b** exon5-7dup. **c** exon13-14dup. **d** exon1-22del. **e** exon5-7dup was confirmed by LR-PCR

Among the 174 patients lacking *BRCA1/2* small pathogenic variants, three unique *BRCA1* LGRs were detected in 5 (2.9%) cases by MLPA assay (Fig. 1a, b, c, d). These include one case with exon5-7dup, two cases with exon13-14dup, and two cases with exon1-22del (Table 2). To our knowledge, these three LGRs have not been reported in Chinese HBOC patients. To confirm MLPA results, we validated exon 5-7dup by designing oligo primers surrounding the putative junction. We obtained a clear and strong 6-8Kb PCR amplicon (Fig. 1e), whose sequence identity was confirmed by Sanger sequencing (data not shown) supporting a tandem duplication event. Overall, *BRCA1* LGRs accounted for 16.1% (5/31) of all patients with *BRCA1* pathogenic variants. No LGR was identified in *BRCA2*. Combining small nucleotide variants and LGRs, we obtained a frequency of 22.5% (49/218) for this cohort, with *BRCA1* LGRs accounting for 10.2% (5/49) of all *BRCA1/2* pathogenic variants.

**Table 2:** *BRCA1* LGRs in 174 familial breast and/or ovarian cancer patients

Family ID	Mutation	Sex of proband	Phenotype proband (age Dx)	Tumor type	IHC	Familial history of breast cancer and ovarian cancer (age Dx)	Other cancers in the family (age Dx)
147	Exon5-7dup	Female	43	IDC	ER−/PR−/HER2-	S OC (48)	None
10	Exon13-14dup	Female	29	MpBC	ER−/PR−/HER2-	M BC(57), PGM BC	None
213	Exon13-14dup	female	33	IDC	ER+/PR−/HER2-	PA OC(53) and BC(56), MGM BC (52)	None
113	Exon1-22del	Female	45 (R), 50 (L)	BMC (R), IDC (L)	R: ER−/PR−/HER2+ L: NA	S BC (42) and OC (45)	F EC (71), MU BlaC (71), MB Leu
203	Exon1–24(part)del	Female	39	MBC	ER−/PR−/HER2-	M BC (50), MS BC (43)	None

*LGRs* Large genomic rearrangements, *Dx* diagnosis, *IHC* immunohistochemistry, *dup* duplication, *del* deletion, *R* right, *L* left, *MpBC* micropapillary breast cancer, *BMC* breast mucinous carcinoma, *IDC* invasive ductal carcinoma, *MBC* medullary breast carcinoma, *BC* breast cancer, *OC* ovarian cancer, *EC* esophageal carcinoma, *BlaC* bladder carcinoma, *Leu* leukemia, *M* mother, *PGM* paternal grandmother, *S* sister, *MS* maternal sister, *PA* paternal aunt, *MGM* maternal grandmother, *F* father, *MU* maternal uncle, *MB* maternal brother

### Disease pathology associated with *BRCA1* LGRs

**Fig. 2:**
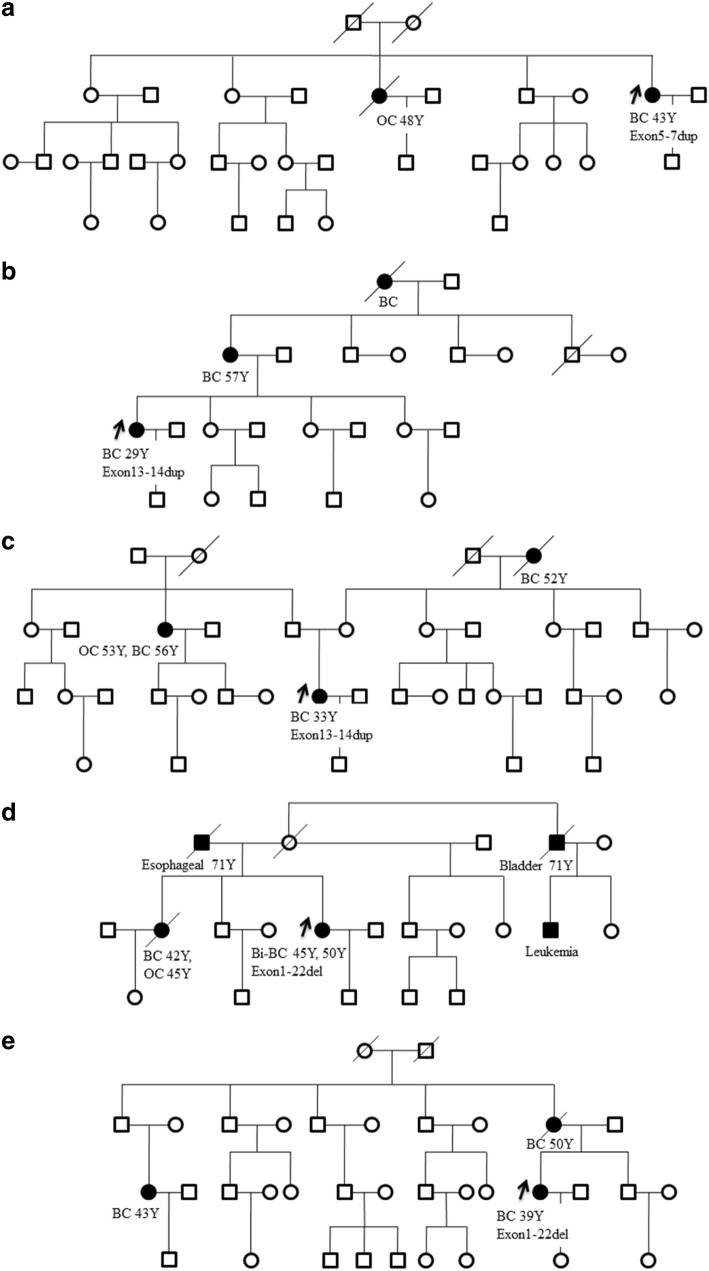
Pedigree of families with proband carrying *BRCA1* LGRs. **a** 147th family with proband carrying exon5-7dup. **b** 10th family with proband carrying exon13-14dup. **c** 213th family with proband carrying exon13-14dup. **d** 113th family with proband carrying exon1-22del. **e** 203th family with proband carrying exon1-22del. (BC, breast cancer; OC, ovarian cancer; Bi-BC, bilateral breast cancer)

The characteristics and familial cancer history of patients with *BRCA1* LGRs were listed in Table 2 and their family pedigrees were shown in Fig. 2. In the five breast cancer patients with *BRCA1* LGRs, the most common tumor type was invasive ductal carcinoma. Moreover, Micropapillary carcinoma, mucinous carcinoma as well as medullary carcinoma was found in these patients with *BRCA1* LGRs. Although triple negative (ER−/PR−/HER2-) subtype was the most common subtype, luminal subtype (ER+) and HER2-overexpression subtype (ER−/PR−/HER2+) were also exited in patients with *BRCA1* LGRs.

## Discussion

In this study, we performed nucleotide sequencing for 218 unrelated FBOC patients living in Eastern China and observed a 20.2% overall pathogenic variant frequency for *BRCA1/2* genes. MLPA assay on the patients lacking small pathogenic variants (174 of 218) further identified 3 unique LCRs in 5 patients, increasing the total *BRCA1/2* pathogenic variant frequency to 22.5%. All of the three *BRCA1* LGRs were not previously reported by Chinese population studies [15–17]. Interestingly, no LGR was identified in *BRCA2* gene within our cohort, consistent to the knowledge that LGRs are more frequently observed in *BRCA1* than *BRCA2*. It was revealed that *Alu*-mediated unequal homologous recombination could be the most common mechanism of LGRs found in *BRCA1/2*, as 72.84% (59/81) and 52.94% (9/17) LGRs in *BRCA1* and *BRCA2* respectively were mediated by this manner [20]. The reason behind higher LGRs frequency in *BRCA1* than in *BRCA2* might be due to the higher Alu density (41.5%) in the *BRCA1* gene than in the *BRCA2* gene [21].

It has been reported that frequency of LGRs ranges from approximately 6–27% of all detected *BRCA1* pathogenic variants, and *BRCA2* LGRs play a less role in hereditary breast cancer patients [20]. Thirty five out of three hundreds (12%) of *BRCA1/2*-sequencing negative familial breast cancer patients from non-Ashkenazi Jewish in US were found carrying *BRCA* LCRs, with 10% (31/300) LCRs detected in *BRCA1* and 1% (4/300) detected in *BRCA2*, respectively [22]. A nationwide study conducted in South Korea showed that LGRs were detected in 3.7% (3/81) of patients bearing *BRCA1/2* pathogenic variants and 7.5% (3/40) of patients bearing only *BRCA1* pathogenic variants [23]. A large sample screening of high-risk breast cancer patients from Hong Kong showed that LGRs accounted for 6.67% (8/120) of all *BRCA1/2* pathogenic variants, involving 8.77% (5/57) of *BRCA1* and 4.76% (3/63) of *BRCA2*, respectively [15]. In our present study, *BRCA1* LGRs account for 16.1% (5/31) of all *BRCA1* pathogenic variants and 10.2% (5/49) of all *BRCA1/2* pathogenic variants. For 174 cases with negative sequencing results, five (2.9%) were identified with *BRCA1* LCRs.

## Conclusions

To conclude, our study has provided evidence that *BRCA1/2* LGRs contribute significantly to the development of HBOC in Chinese mainland population and LGRs screening should be taken into consideration in hereditary breast cancer consulting. It is imperative that the frequency and spectrum of *BRCA1/2* should be investigated in the context of both small nucleotide variants and LGRs.

## Additional files

Additional file 1: **Table S1.** Primers for entire coding exons and intron-exon boundaries of *BRCA1*. (DOCX 30 kb)

Additional file 2: **Table S2.** Primers for entire coding exons and intron-exon boundaries of *BRCA2*. (DOCX 31 kb)

## Data Availability

The datasets used and/or analyzed during the current study are available from the corresponding author on reasonable request.

## Abbreviations

BC: Breast cancer
IHC: Immunohistochemistry
LGRs: Large genomic rearrangements
MAQ: Multiplex amplicon quantification
MLPA: Multiplex ligation dependent probe amplification
NGS: Next generation sequencing
OC: Ovarian cancer
PCR: Polymerase chain reaction
