# Supplementary material for: Comprehensive mutation detection of BRCA1/2 genes reveals large genomic rearrangements contribute to hereditary breast and ovarian cancer in Chinese women
Source: BMC Cancer. 2019 Jun 7;19:551. doi: 10.1186/s12885-019-5765-3 (PMC6555923; doi:10.1186/s12885-019-5765-3)
Supplement: Supplementary file 1 — Table S1. Primers for entire coding exons and intron-exon boundaries of BRCA1. (DOCX 30 kb) [file 12885_2019_5765_MOESM1_ESM.docx]

sTable 1 Primers for entire coding exons and intron-exon boundaries of *BRCA1*

Exons Primer sequence (5'-3')* Tm (°C) Size (bp)

| Exon2 | F: GGACGTTGTCATTAGTTCTTTG | 55 | | 430 |
| --- | --- | --- | --- | --- |
|  | R: GTGGATGGAGAACAAGGAATC |  | |  |
| Exon3 | F: TCCTGACACAGCAGACATTTA | 60 | | 338 |
|  | R: TTGGATTTTTCGTTCTCACTTA |  | |  |
| Exon5 | F: GCTTGTAATTCACCTGCCAT | 58 | | 269 |
|  | R: TTCCTACTGTGGTTGCTTCC |  | |  |
| Exon6 | F: AGGTTTTCTACTGTTGCTGCAT | 55 | | 306 |
|  | R: AAAAGGTCTTATCACCACGTCA |  | |  |
| Exon7 | F: CACAACAAAGAGCATACATAGG | 60 | | 269 |
|  | R: AGAAGAAGAAGAAAACAAATGG |  | |  |
| Exon8 | F: TTTCCAGGCATCATACAT | 50 | | 377 |
|  | R: CTCACCATAGGGCTCATA |  | |  |
| Exon9 | F: TGCCACAGTAGATGCTCAGT | 57 | | 292 |
|  | R: CACATACATCCCTGAACCTAAA |  | |  |
| Exon10 | F: TGGTCAGCTTTCTGTAATCG | 60 | | 242 |
|  | R: GTATCTACCCACTCTCTTTTCAG |  | |  |
| Exon11a | F: TAGCCAGTTGGTTGATTTCC | 62 | | 477 |
|  | R: CTCACACAGGGGATCAGCATTC |  | |  |
| Exon11b | F: CAACATAACAGATGGGCTGGAAG | 66 | | 296 |
|  | R: ACGTCCAATACATCAGCTACTTTGG |  | |  |
| Exon11c | F: GGTTCTGATGACTCACATGATGGG | 65-58 | | 460 |
|  | R: TCATCACTTGACCATTCTGCTCC |  | |  |
| Exon11d | F: GAGCCACAGATAATACAAGAGCGTC | 60 | | 273 |
|  | R: GCAGATTCTTTTTCGAGTGATTCTATTGGG | | |  |
| Exon11e | F: ATCAGGGAACTAACCAAACGGAG | | 56 | 502 |
|  | R: CAGCTCTGGGAAAGTATCGCTG | |  |  |
| Exon11f | F: GCAACTGGAGCCAAGAAGAG | | 58 | 647 |
|  | R: CCAGAGTGGGCAGAGAATGT | |  |  |
| Exon11g | F: TGAACTTGATGCTCAGTATTTGC | | 56 | 345 |
|  | R: AGTCCAGTTTCGTTGCCTCT | |  |  |
| Exon11h | F: TAAGCCAGTTGATAATGCCA | | 60 | 430 |
|  | R: TTTTGGCCCTCTGTTTCTAC | |  |  |
| Exon11k | F: ACTAATGAAGTGGGCTCCAG | | 57 | 445 |
|  | R: CCAAATGTGTATGGGTGAAA | |  |  |
| Exon11m | F: CAGCCTATGGGAAGTAGTCATGC | | 58 | 550 |
|  | R: TCAAGAAAGGATCCTGGGTGTT | |  |  |
| Exon11n | F: CGTTGCTACCGAGTGTCTGTCTAAG | | 63-56 | 438 |
|  | R: GTGCTCCCCAAAAGCATAAA | |  |  |
| Exon12 | F: GTCCTGCCAATGAGAAGAAA | | 60 | 265 |
|  | R: TGTCAGCAAACCTAAGAATGT | |  |  |
| Exon13 | F: GGTGATTTCAATTCCTGTGC | | 58 | 373 |
|  | R: AAATGTTGGAGCTAGGTCCTTAC | |  |  |
| Exon14 | F: CTAACCTGAATTATCACTATCA | | 60 | 313 |
|  | R: GTGTATAAATGCCTGTATGCA | |  |  |
| Exon15 | F: TTGCCAGTCATTTCTGATCT | | 54 | 482 |
|  | R: AAACCTTGATTAACACTTGAGC | |  |  |
| Exon16 | F: AATTCTTAACAGAGACCAGAAC | | 60 | 449 |
|  | R: AAAACTCTTTCCAGAATGTTGT | |  |  |
| Exon17 | F: GTGTAGAACGTGCAGGATTG | | 60 | 263 |
|  | R: TCGCCTCATGTGGTTTTA | |  |  |
| Exon18 | F: GGCTCTTTAGCTTCTTAGGAC | | 60 | 352 |
|  | R: GAGACCCATTTTCCCAGCATC | |  |  |
| Exon19 | F: CTGTCATTCTTCCTGTGCTC | | 60 | 249 |
|  | R: CATTGTTAAGGAAAGTGGTGC | |  |  |
| Exon20 | F: CCATGTTGGTCAGACTGGTG | | 58 | 346 |
|  | R: CTGCAAAGGGGAGTGGAATA | |  |  |
| Exon21 | F: CATCAGGTGGTGAACAGAA | | 55 | 242 |
|  | R: TAAGACAAAGGCTGGTGC | |  |  |
| Exon22 | F: TCCCATTGAGAGGTCTTGCT | | 60 | 297 |
|  | R: GAGAAGACTTCTGAGGCTAC | |  |  |
| Exon23 | F: GGCAGAGGTGGCAGTGAGC | | 60 | 469 |
|  | R: TGCCCGGCCTTGATTTATG | |  |  |
| Exon24 | F: TGGAGTCGATTGATTAGAGC | | 60 | 311 |
|  | R: AGCCAGGACAGTAGAAGGAC | |  |  |

*F: forward; R: reverse
