# Supplementary material for: Comprehensive mutation detection of BRCA1/2 genes reveals large genomic rearrangements contribute to hereditary breast and ovarian cancer in Chinese women
Source: BMC Cancer. 2019 Jun 7;19:551. doi: 10.1186/s12885-019-5765-3 (PMC6555923; doi:10.1186/s12885-019-5765-3)
Supplement: Supplementary file 2 — Table S2. Primers for entire coding exons and intron-exon boundaries of BRCA2. (DOCX 31 kb) [file 12885_2019_5765_MOESM2_ESM.docx]

sTable 2 Primers for entire coding exons and intron-exon boundaries of *BRCA2*

Exons Primer sequence (5'-3')* Tm (°C) Size (bp)

| exon2 | F: AGGAGATGGGACTGAATTAG | 50/52 | 311 |
| --- | --- | --- | --- |
|  | R: GTGACGTACTGGGTT |  |  |
| exon3 | F: CTTTAACTGTTCTGGGTCAC | 50 | 480 |
|  | R: ATTATTTTCATCGTCTCCAT |  |  |
| exon4 | F: CCTCTTCTTACAACTCCCTA | 50 | 360 |
|  | R: GATCTTCTACCAGGCTCTTA |  |  |
| exon5/6 | F: ATACACGGTTTCCAGCAGC | 55 | 491 |
|  | R: CAGGGCAAAGGTATAACGC |  |  |
| exon7 | F: CTAGCATTCTGCCTCATACA | 50 | 289 |
|  | R: TTATCAACCTCATCTGCTCTT |  |  |
| exon8 | F: ATGTAATCAAATAGTAGATGTGC | 50 | 214 |
|  | R: ATTTAACAAGGCATTCCAA |  |  |
| exon9 | F: GATAACTGAAATCACCAAAAG | 50 | 297 |
|  | R: GAGCAAGACTCCACCTCA |  |  |
| exon10a | F: AGAACAGGAGAAGGGGTGAC | 55 | 571 |
|  | R: CACAGGCCAAAGACGGTA |  |  |
| exon10b | F: GAAGTGGAACCAAATGATACT | 50/52 | 588 |
|  | R: GGAGTCCTCCTTCTGTGAG |  |  |
| exon10c | F: CTTCTTCATTTCAGGGTATC | 48/50 | 552 |
|  | R: GTATACAGATGATGCCTAAGA |  |  |
| exon11a | F: CTGTGCCCAAACACTACC | 52/55 | 457 |
|  | R: TTCTGGGATTGAAAGTCAG |  |  |
| exon11b | F: TTAACTAGCTCTTTTGGGACA | 55 | 532 |
|  | R: TGGCAACAGCTCAACG |  |  |
| exon11c | F: GACAAGCTCAAAGGTAACAA | 50/53 | 546 |
|  | R: CAAGGAGATGTCCGATTT |  |  |
| exon11d | F: AAGCAACCCAAGTGTCAA | 52/55 | 532 |
|  | R: AGTTCTGTAATTTCTGCCTTT |  |  |
| exon11e | F: TGTTATTTTCCAAGCAGGA | 50/52 | 617 |
|  | R: TTGTAATATCAGTTGGCATTT |  |  |
| exon11f | F: TGCAGAGGTACATCCAATAAG | 55 | 429 |
|  | R: GCTTTCGCAACTTCCAA |  |  |
| exon11g | F: AAACGGACTTGCTATTTACT | 50 | 483 |
|  | R: CATCACGTTCGGGTTG |  |  |
| exon11h | F: CTAAGTTATGAGGAAACAGACA | 50 | 559 |
|  | R: TGACTGAATAAGGGGACTG |  |  |
| exon11i | F: GACTGTGGTGCCACCTAAG | 55 | 578 |
|  | R: GGTATGCATTTGCATCTTTTA |  |  |
| exon11j | F: GATTCTGGTATTGAGCCAGTA | 55 | 457 |
|  | R: AAACCTTATGTGAATGCGTG |  |  |
| exon11k | F: CGAGGCATTGGATGATTC | 55 | 561 |
|  | R: TCTAACACTCCCTTAACTTTGTG |  |  |
| exon11l | F: TCAGACCAGCTCACAAGAG | 54/55 | 660 |
|  | R: TGTCAGTTCATCATCTTCCA |  |  |
| exon11m | F: AGAACAGGCTTCACCTAAAA | 54/55 | 432 |
|  | R: TCCCCCAAACTGACTACAC |  |  |
| exon12 | F: ATCCTGTTTAGACCCTGTTA | 55 | 441 |
|  | R: GTCTGTAATCCCAGCACTT |  |  |
| exon13 | F: GAGTTATTTGGTGCATAGTCAT | 54/55 | 536 |
|  | R: TCGGAGCAATTTCCTTAA |  |  |
| exon14 | F: AGAGGGTCTGCAACAAAGG | 55 | 566 |
|  | R: AACGGAAATATCTAACTGAAAGG |  |  |
| exon15 | F: AGAGACAGGGTTTCTCCAT | 50 | 558 |
|  | R: GATCACTTTAGCAGGATGAG |  |  |
| exon16 | F: ATGGCAGATTTAGCAGGA | 50 | 501 |
|  | R: AGAAGAAAGAGGGATGAGG |  |  |
| exon17 | F: CTGGGAGTATAGGCATGAGC | 55 | 599 |
|  | R: GGGAAAGAGGGAAGCAAG |  |  |
| exon18a | F: CACTATTTGGGGATTGCTAA | 55 | 498 |
|  | R: GACCAACTGTCAGTCTGCC |  |  |
| exon18b | F: AACTTACAGATGGGTGGTA | 50 | 492 |
|  | R: CCAGAGTGGAATAGGGA |  |  |
| exon19 | F: TACTGTCTTACTAATCTTCCTAAGA | 50 | 383 |
|  | R: GACCGAAACTCCATCTCA |  |  |
| exon20 | F: CCCAAAGTTCTGGGATTAC | 50/54 | 454 |
|  | R: CAAATGGCTTAGACCTGATAT |  |  |
| exon21 | F: CTCCCTTCTTTGGGTGTT | 55 | 422 |
|  | R: AACCCTCCCATCCTCTACT |  |  |
| exon22 | F: AGTTTGAGGCACCTGAGAATA | 55 | 503 |
|  | R: TTGTGGGCATTAGTAGTGGA |  |  |
| exon23/24 | F: CCACTACTAATGCCCACAAA | 55 | 603 |
|  | R: CAACTGGTAGCTCCAACTAATC |  |  |
| exon25 | F: GAGTTTCCTTTCTTGCATC | 50 | 389 |
|  | R: TTCCTTGATACTGGACTGTC |  |  |
| exon26 | F: GCATCGGCATGTTTGAC | 55 | 446 |
|  | R: TAACTATACTTACAGGAGCCACA |  |  |
| exon27a | F: TAGGAGTTAGGGGAGGG | 50 | 515 |
|  | R: GCAAGTTCTTCGTCAGC |  |  |
| exon27b | F: AAATACGAAACACCCATAAA | 50/52 | 383 |
|  | R: GGAAAGGTTAAGCGTCAA |  |  |

*F: forward; R: reverse
